# A Study on Ultraviolet Protection of 100% Cotton Knitted Fabric: Effect of Fabric Parameters

**DOI:** 10.1155/2014/506049

**Published:** 2014-05-18

**Authors:** C. W. Kan

**Affiliations:** Institute of Textiles and Clothing, The Hong Kong Polytechnic University, Hung Hom, Kowloon, Hong Kong

## Abstract

The effect of fabric parameters such as weight, thickness, and stitch density on the ultraviolet (UV) protection of knitted fabrics was studied. Different knitting structures such as plain, pineapple, lacoste, and other combinations of different knitting stitches of knit, tuck, and miss as well as half milano, full milano, half cardigan, full cardigan, 1 × 1 rib, and interlock were prepared. Experimental results revealed that weight was the most important factor that affected UV protection while thickness and stitch density were not the leading factor in determining UV protection.

## 1. Introduction

Researches prove that ultraviolet radiation (UVR) from the sun could be a primary cause of skin cancer [1, 2]. The number of skin cancer cases found has been increasing around the world in the recent years, including both nonmelanoma and melanoma skin cancers [3]. In terms of local health issue, the number of nonmelanoma skin cancers found is also increasing according to the statistics from the Hong Kong Cancer Registry of Hospital Authority. Nonmelanoma skin cancer is ranked the eighth among the top ten cancers in Hong Kong in terms of incidence [4]. As a result, the adverse impact caused by overexposure to UVR has increased the public awareness of the need to adopt personal UV protective strategies such as the use of sunscreens on the parts of body that are exposed to the sun [5]. Apart from sunscreen and shading, wearing textile garments could be a practical solution to avoid the contact of skin and UVR [6–12]. Many researchers have studied various fabric parameters that influence UVR transmission including fiber composition [13–16], fabric construction [16–21], yarn twist [22], thickness [13, 15, 23], weight [23], wetness or moisture content [24, 25], stretch or extensibility [24, 26], chemical treatment or additives, and coloration [27–32]. However, most of the studies have concentrated on the above fabric parameters with woven fabrics only, whereas there have been few studies concerning knitted fabrics. In summer time, there is a higher chance of UVR exposure in terms of intensity and duration while knitted garment is much more popular in that season. Therefore, the aim of this study is to examine the ability of UV protection of knitted fabric in terms of fabric parameters. The ultraviolet protection factor (UPF) will be used as a measuring parameter of the UV protection.

## 2. Materials and Methods

### 2.1. Materials

Ten types of 100% cotton yarns (provided by Central Textiles (H.K.) Ltd.) were used and their specifications are summarized in Table 1. Each yarn type was used to produce different knitted structures including nine single knitted structures and six double knitted structures with the use of Stoll CMS 822 14G computer flat knitting machine. For the nine single knitted structures, three of them were general types including plain knit (single jersey), pineapple, and lacoste, while the other six of them were different combinations of knit, tuck, and miss stitches including (i) knit and tuck with ratio 1 : 1, (ii) knit and miss with ratio 1 : 1, (iii) knit and tuck with ratio 2 : 2 along the wale direction, (iv) knit and miss with ratio 2 : 2 along the wale direction, (v) knit and tuck with ratio 2 : 2 along the course direction, and (vi) knit and miss with ratio 2 : 2 along the course direction. The notations of the nine single knitted structures are shown in Table 2. For the double knitted structures, the six structures chosen were half milano, full milano, half cardigan, full cardigan, 1 × 1 rib, and interlock which were shown in Table 3.

After preparing the knitted fabrics, combined scouring and bleaching process was carried out as pretreatment and the treatment bath, containing Sandopan DTC (5 g/L), sodium hydroxide (10 g/L), stabilizer AWN (1 mL/L), and 35% hydrogen peroxide (25 mL/L), was prepared. Fabric samples were padded with the liquor at 30°C until 100% wet pickup. Those padded fabric samples were steamed for 30 minutes at 102–105°C and then were rinsed thoroughly in hot and cold water. Finally, the fabric samples were laid flat and air-dried completely in conditioning room with relative humidity of 65 ± 2% and temperature of 20 ± 2°C in order to avoid shrinkage during drying. After drying, the fabric samples were conditioned at relative humidity of 65 ± 2% and temperature of 20 ± 2°C for at least 24 hours before use.

### 2.2. UPF Measurement

In this study,* in vitro* approach was used to measure the protection ability of cotton knitted fabrics instead of* in vivo* one since it was able to provide a simple method of rating the UV protective abilities of fabrics by using relatively low-cost procedures. The* in vitro* measurement of fabric protective ability was conducted with a spectrophotometer (Varian Cary 300 UV-visible spectrophotometer) in accordance with the AS/NZS 4399 standard. Ultraviolet protective factor (UPF) was used in this study as a quantitative indicator to represent the UV protective capabilities of knitted fabrics from sunburn. The UPF was calculated by

(1) $$\begin{array}{c} \text{UPF}=\frac{\sum_{290}^{400}E_{\lambda }\cdot S_{\lambda }\cdot \Delta_{\lambda }}{\sum_{290}^{400}E_{\lambda }\cdot S_{\lambda }\cdot T_{\lambda }\cdot \Delta_{\lambda }}, \end{array}$$

where *S*_*λ*_ is the solar spectral irradiance (in W · m^−2^ · Nm^−1^), *E*_*λ*_ is the erythemal spectral effectiveness from CIE (1987), *T*_*λ*_ is the spectral transmission through the textile, Δ_*λ*_ is the bandwidth (in nm), and *λ* is the wavelength (in nm).

### 2.3. Measurements of Knitted Fabrics Parameters

Fabric weight per unit area (g/m^2^) was measured according to ASTM D3776-1996. Fabric thickness was measured by the fabric thickness tester (Hans Baer AG CH-Zurich Telex 57767) according to ASTM D1777-1996. With the measurement of the fabric weight and thickness, the weight-to-thickness (*W*/*T*) ratio was obtained as shown in the following:

(2) Weight-to-thickness  ratio(WT) =Fabric  weight  (g/m2)Fabric  thickness  (mm).

Course density is the number of visible loops per unit length measured along a wale while the wale density is the number of visible loops per unit length measured along a course. Stitch density is the multiple of course density and wale density as shown in the following:

(3) Stitch  density  (N) =Course  per  inch  (cpi)×Wales  per  inch  (wpi).

Both the course density and the wale density were measured by visual observation. The number of courses and wales were counted in a 2.54 cm fixed length under a magnifying glass with aid of a pointed metal needle.

## 3. Results and Discussion

From previous researches, it is assumed that thickness and weight are the factors contributing in the determination of UPFs of knitted fabric [33, 34]. The value of UPF increases with increase in fabric density and thickness for similar construction. In order to investigate the relationship between UPF, weight, and thickness, two approaches were used, either the change of UPF within same structure or on different knitting structures.

In this study, the weight, thickness, and weight-to-thickness (*W*/*T*) ratio for single and double knitting structures are collected in order to analyze the relationship between them and UPF, respectively. Correlation analysis will be used and the purpose is to evaluate the relative impact of a predictor variable on a particular outcome [35]. The correlation coefficient (*R*) is used to measure the monotonic relationship between two variables by increasing the value of the other variable. The relationship of the two variables could be explained by correlation coefficient (*R*) according to Table 4.

Moreover, the coefficient of determination (*R* ^2^) with a range of 0–1 was also used in the correlation analysis. Coefficient of determination (*R* ^2^) was the fraction of the variability in one variable that can be explained by the variability in other variable through their linear relationship, or vice versa [35]. It could be used to determine how well the future outcomes would be likely to be predicted by the model.

### 3.1. Effect of Fabric Weight on UPF

The statistical relationship between fabric weight and UPF is calculated and summarised in Table 5. The correlation coefficient *R* and coffeicient of determination (*R* ^2^) give the overall goodness of fit measures of the linear regression model between fabric weight and UPFs in single knitted structures.

For single knitted structures, it is found that all of the *R* values of single knitted structures are positive which means the relationship of fabric weight is positively related to the UPF. However, the magnitude of *R* value varies greatly among single knitted structures. For example, the *R* values of pineapple and plain structures are very low, only 0.16 and 0.28, respectively, which means that the relationship of fabric weight and UPF in pineapple and plain structures is weakly positive. On the other hand, the *R* value of KM22C is extremely high which is close to 1 which means that the relationship between fabric weight and UPF of KM22C structure is perfectly positive.

Moreover, the *R* ^2^ of KM22C structure between UPF and fabric weight is 0.94 which means about 94% of UPF of KM22C structure could be explained by fabric weight. Like the result in *R* value, pineapple structure had the least *R* ^2^ value, only 0.02. In pineapple structure, fabric weight could not be used to predict the UPF since only 2% of the data of UPF could be explained by fabric weight. It is found that the knit-tuck-miss structures, including KT11, KM11, KT22C, KM22C, KT22W, and KM22W, generally have higher *R* and *R* ^2^ values than the plain and pineapple structures.

For the double knitted structures, it is found that the correlation between UPF and fabric weight of double knitted structures varies greatly. Among all double knitting structures, half milano is the only structure which shows the result of slightly negative relationship between the fabric weight and UPF since it has a negative *R* value (−0.15) and the slope of linear regression line is also negative. Moreover, the correlation between the two variables of half milano is also very weak since the *R* ^2^ value is also extremely small, only 0.02. This means only 2% of the UPF data in half milano structure could be explained by fabric weight. The other double knitted structures except half milano are found to have positive relationship between fabric weights and UPF. Half cardigan has the highest *R* value, 0.94, which means the correlation between the two variables is strongly positive. The *R* ^2^ value of half cardigan is also the highest while all the others are comparatively quite low.

In order to have a more general review on the effect of fabric weight on UPF in terms of knitted structure, the relationship between mean fabric weights and mean UPFs of all yarn types in terms of knitted structures was also investigated. The result of mean fabric weight and mean UPFs is shown in Table 5. From Table 5, it is found that all the mean fabric weights of double knitted structures are greater than that of single knitted structures. Since double knitted fabrics are manufactured with two needle beds, two layers are formed. Therefore, the double knitted fabric generally had a greater mean fabric weight. As a result, it shows that the fabric weight is one of the factors that affected the difference of the UPF between single and double knitted structures.

For the single knitted structure, the results of mean fabric weight and mean UPF of single knitted structures in Table 5 show that, for the maximum and minimum points, the KT22W structure has the lowest mean fabric weight and mean UPF while KM22W has the highest mean fabric weight and mean UPF. In general, there is an increasing trend of the fabric weight with increasing UPF. It is obvious that the knit-and-miss structures (KM11, KM22W, and KM22C) have generally higher mean fabric weight than the other single knitted structures. It is due to the effect of miss stitches which would make the fabric become narrower and bulkier. For double knitted structures, there is a general increasing trend of UPF with the increasing fabric weight from half milano to interlock. Interlock has the highest mean fabric weight as well as mean UPF. However, the UPF of some double knitted structures could not fully be explained by the fabric weight. Full cardigan has the lowest mean UPF but the mean fabric weight of full cardigan is higher than that of 1 × 1 rib. Half cardigan also has a higher fabric weight than 1 × 1 rib, but the UPF is lower. In order to further investigate the relationship between mean fabric weight and mean UPFs, the statistical relationship of single and double knitted structures is listed in Table 5.

From Table 5, it is found that there is a strongly positive relationship between the mean fabric weights and mean UPFs for single and double knitted structures. Both the *R* values of single and double knitted structures are very high and higher than 0.8. According to Table 5, the correlation between mean fabric weight and mean UPF of both single and double knitted structures is strongly positive. Moreover, the *R* ^2^ values of single and double knitted structures are also quite high which are 0.91 and 0.88, respectively, which means that 91% and 88% of mean UPF of single and double knitted structures can be represented by the mean fabric weight, respectively. Generally speaking, the mean UPFs of different knitted structures are also strongly positively correlated with the mean fabric weights since the *R* value is 0.89 which is greater than 0.8. This means 80% of the mean UPF can be explained by the mean fabric weight as the *R* ^2^ value was 0.80. Fabric with higher weight would provide more fibers and yarns in the fabric structure when compared with lighter ones, and therefore UV radiation is scattered and becomes more difficult to penetrate through the fabric and contact the skin. In conclusion, fabric weight is one of the factors that are able to affect the UPF. The results are generally in agreement that, with heavy fabric weight, the space between yarns is smaller. The heavier fabric weight allows less UV radiation to penetrate and therefore has higher UPF values [15].

### 3.2. Effect of Fabric Thickness on UPF

The statistical relationship between fabric thickness and UPF is shown in Table 6.

It is found in Table 6 that all knitted structures have different degrees of positive relationship between the fabric thickness and UPF except for the pineapple structure (both the *R* and *R* ^2^ values were 0.00) which means there is no association between these two variables in pineapple structure. The *R* and *R* ^2^ values of the other structures vary as well. Only KT22 M has a relatively higher *R* value (0.71) but the *R* ^2^ value is only 0.51, which means only around half of the UPF data in KM22C structure is correlated with the fabric thickness. All the *R* ^2^ values in other structures are smaller than 0.5.

In order to have a more general review on the effect of fabric thickness on UPF in terms of knitted structure, the relationship between mean fabric thicknesses and mean UPFs of all yarn types in terms of knitted structures was also investigated. The result of mean fabric weight and mean UPFs is shown in Table 6. From Table 6, it is found that all the mean thicknesses of double knitted structures are greater than that of single knitted structures. It is because the double knitted fabrics are composed of two layers which are formed by two needle beds in the knitting process. Moreover, all the mean UPFs of double knitted structures are also greater than that of single knitted structures. There is a general trend that thicker fabric has a higher UPF. All the knit and knit-with-tuck structures have increasing thickness with increasing UPF except for KT22W. KT22W has a high mean thickness but the lowest mean UPF. The knit-with-miss structures (KM11, KM22W, and KM22C) also follow the trend that mean UPF increases with mean thickness. However, lacoste has a higher thickness than KM11 but lower UPF. There are slight differences in mean thickness but large differences in mean UPF between knit-and-miss structure and that of knit or knit-and-tuck structures.

The mean thickness of the half milano, full milano, 1 × 1 rib, and interlock structures increases with the increasing mean UPFs. Interlock structure has the highest thickness and highest mean UPF. 1 × 1 rib has the second low mean UPF and mean thickness. However, the cardigan structures are once again the exceptions since they have significantly high mean thickness but very low mean UPF, especially for half cardigan. Full cardigan has a very high mean thickness but the mean UPF is very low; half cardigan also had the highest mean thickness, but the mean UPF is quite low. The situation of cardigans is similar and even more significant when compared with that of the mean weight. It is found that mean thickness is not the dominant factor that affects the mean UPF in double knitted structure. Structure and type of stitches play a more important role in determination of the UPF in double knitted structure.

Moreover, linear regression method is also used to find the statistical relationship between mean thickness and mean UPF of single and double knitted structures and the results are shown in Table 6. From Table 6, it is found that the relationship between the mean fabric thickness and mean UPF for single and double knitted structures is positive since the *R* value and slope of regression lines are positive. According to Table 6, the *R* value of single knitted structure is 0.62 which means fabric thickness was moderately positive to the mean UPF in single knitted structure. In double knitted structure, the *R* value is lower (0.37) which means the correlation between mean fabric thickness and mean UPF in double knitted structure is slightly positive. Moreover, the *R* ^2^ values of single and double knitted structure are also quite low which were 0.38 and 0.13, respectively.

### 3.3. Effect of Weight-to-Thickness Ratio on UPF

Apart from the weight and thickness themselves, the ratio between these two parameters was also taken into consideration. The weight-to-thickness parameter represented the relative weight of each structure and can be calculated by (2). Correlation analysis is used to investigate the relationship between weight-to-thickness ratio and UPF. The statistical relationship is calculated and shown in Table 7.

In Table 7, all the single knitted structures have positive relation between the mean *W*/*T* ratio and mean UPF. For double knitted structures, only 1 × 1 rib structure has slightly negative correlation between the two variable and all the other double knitted structures have different degrees of positive correlation.

Nevertheless, most of the *R* ^2^ values of knitted structures are quite low. Seven out of fifteen structures have a *R* ^2^ value less than 0.2 and only three structures, KM22C, full milano, and interlock structures, have *R* ^2^ values greater than 0.5. In order to have a more general review on the effect of fabric weight-to-thickness ratio UPF in terms of knitted structure, the relationship between mean fabric weight-to-thickness ratio and mean UPFs is investigated and the results are listed in Table 7. From Table 7, it is found that, similar to the result of mean weight and mean thickness, most of the mean *W*/*T* ratios of double knitted structures are greater than that of single knitted structures, except that full cardigan has lower mean *W*/*T* ratio than KM22W.

In the single knitted structures, some structures have gradually negative proportional relationship between the mean *W*/*T* ratio and mean UPF. The UPF increases with the decreasing *W*/*T* ratio. However, plain knit has the highest mean *W*/*T* ratio but the mean UPF is the second lowest one. Some exceptional cases are found; for example, KM22W has the highest mean UPF while the *W*/*T* ratio is moderate. KT22W is a special case since it has the lowest UPF and the lowest *W*/*T* ratio and the relationship is not inversely related. Moreover, the *W*/*T* ratio of KM11 is higher than that of lacoste yet the UPF is higher. This could be explained by difference of stitches formation; the use of miss stitches for KM11, KM22C, and KM22W. Thus, no concrete conclusion could be drawn for the relationship of single knitted structures because other parameters like effect of miss stitch also were more dominant than the *W*/*T* ratio on the influence on UPF.

For the double knitted structure, it is found that the mean *W*/*T* ratios for double knitted structures have positive relationship with the mean UPF; that is, the mean UPF increases with the increasing mean *W*/*T* ratio. Full cardigan structure has the lowest mean *W*/*T* ratio and the least mean UPF while interlock has the highest *W*/*T* ratio and the highest mean UPF.

Moreover, linear regression method is also used to find the statistical relationship between mean *W*/*T* ratio and mean UPF of single and double knitted structures as shown in Table 7. From Table 7, it is found that the relationship between the mean *W*/*T* ratio and mean UPF for single and double knitted structures is positive since the *R* values and slope of regression lines were positive. The *R* values of double knitted structures were very high which were 0.92 and greater than 0.8. According to Table 7, they are highly positively correlated within the two variables in double knitted structures. Moreover, the *R* ^2^ value of double knitted structure is also high which is 0.85. This means 85% of the data are found to be related to mean *W*/*T* ratio. For single knitted structures, both the *R* and *R* ^2^ values are low which means the correlation between mean *W*/*T* and mean UPF in single knitted structures is only slightly positive and there is no determination between these two variables since the *R* ^2^ value was 0.08 and close to zero. Although the *R* and *R* ^2^ values of double knitted structure are high which proves that there is significant determination of mean *W*/*T* ratio in double knitted structures, the single knitted structures do not show a similar result. In conclusion, mean *W*/*T* ratio is one of the determination factors of UPF in double knitted structures only but not single knitted structures.

### 3.4. Effect of Stitch Density on UPF

The parameter which can indicate the change of courses and wales per unit area is called stitch density. Stitch density was the multiple of wale density and course density and in this research calculated by (3).

The stitch density is mainly affected by the machine gauge, yarn parameters like yarn count, and the nature of knitting structure. Ogulata and Mavruz [36] mentioned that the increase of stitch density would result in the decrease of pore size values. Since UPF is dependent on porosity where UPF = 100/porosity, stitch density should also be taken into account in the factor that affected UPFs and this section would investigate the relationship between change of stitch density and the subsequent change of the transmission of UV radiation. Correlation analysis is used to investigate the relationship between stitch density and UPF and the statistical relationship is calculated and shown in Table 8.

In Table 8, it is found that stitch densities of all single knitted structures have a positive relation with UPF except KT22C. Also, stitch densities of all the double knitted structures have positive correlation with UPF, except 1 × 1 rib. For the other structures except 1 × 1 rib and KT22C, the UPFs would increase with the increase of stitch densities. However, the result was not ideal. The *R* values in Table 8 are negative in KT22C and 1 × 1 rib structure which represent that the UPF of samples in these two structures increases with the decrease of stitch density. It means that stitch density is not the only and most determinant factor that affects the relation with the two variables within 1 × 1 rib and KM22C structure.

Moreover in Table 8 the *R* ^2^ values of most knitting structures are comparatively low and eleven out of fifteen structures have a *R* ^2^ value smaller than 0.2. Only pineapple and half cardigan have a coefficient of determination greater than or equal to 0.5. Thus, there is not much relation between stitch density and UPF among individual structures. The change of stitch density among individual structures is not obvious as much as the change of UPF. The results of mean stitch density and mean UPF of different knitting structures are listed in Table 8. From Table 8, when comparing the single knitted structures and double knitted structures, the result of mean stitch density is not like that of weight, thickness, or *W*/*T* ratio. It is found that the mean stitch densities of single knitted structures and double knitted structures are varied. Even single knitted structures like plain structures are able to have high stitch density while double knitted structures such as cardigan structures have results of low stitch density.

There is a trend of increasing mean UPF with mean increasing stitch density in single knitted fabric except for plain and pineapple structures. KT22W has the least mean stitch density and least UPF. KM22W has the highest mean UPF and the second high stitch density. It shows that the UPF is dependent on stitch density in general. The increase of stitch density would lead to smaller pore sizes. The decrease in pore sizes would result in decrease in porosity and hence UPF would increase.

Moreover, when comparing single knitted structures with miss stitches, it is found that the one with miss stitches has a higher UPF. It is due to the effect of miss stitches which would narrow and tighten the fabrics and hence results in higher UPF. For example when comparing KT22W and KM22W, although they have the same tuck or miss ratio and the tuck or miss stitches are located in the same position, it is found that the structure with miss stitches would give a higher UPF.

However, exceptions are found since plain and pineapple structures have a high mean stitch density but low mean UPFs. Plain structure is a regular structure whose stitches are packed close together tightly and hence the stitch density is high. Pineapple structure also has high stitch density because large tuck loops across three courses are knitted.

For double knitted structure, a trend similar to the single knitted structures is found and the mean UPFs increase with the increasing mean stitch density except 1 × 1 rib. Full cardigan has the least mean stitch density and mean UPF while interlock has the greatest mean stitch density and mean UPF. The theory that increase in stitch density would result in smaller porosity and high UPF also applied to most of the double knitted structures except 1 × 1 rib. 1 × 1 rib has a higher mean stitch density but lower mean UPF when compared to those of half cardigan. The reason why 1 × 1 rib has high stitch density is that it has only knit structure and is similar to plain knit.

Therefore, the exceptional cases of plain, pineapple, and 1 × 1 structures mean that although some structures have high stitch density but not have high UV protection abilities. Other factors may play a more important role than stich density in determining the UPF. In order to look into the relationship between mean stitch density and mean UPF on single and double knitted structures, linear regression method is used to analyze the results. From *R* values in Table 8, it is proved that all knitted structures have positive relation between stitch density and UPF, so that, when comparing different knitted structures, those with higher stitch density may have higher UPF. However, the *R* ^2^ values in single knitted structure (0.37) are lower than the double knitted structure (0.68). Therefore, the relation of stitch density and UPF is low in single knitted structure and moderate in double knitted structure. From Table 8, it can generally be summarised that, for knitted fabric structures with knit and miss, higher UPF values would be obtained than with knit and tuck structure. The miss loops would pull the knit loops closer to each other and give the fabric higher stitch density. Meanwhile, the miss loop floats at the back of the fabric and therefore less UV radiation can pass through the fabric, resulting in higher UPF value [37].

## 4. Conclusion

The effects of different fabric parameters on UPF are discussed from two aspects: among individual structures and on different structures. Linear regression model is helpfully used to examine their relationship. The effect of fabric weight, thickness weight-to-thickness ratio, and stitch density on UPF was found and comparison was made among these three fabric parameters. The effects of stitch density on UPF was discussed. It is found that weight was the most dominant factor that affects the UPF on different knitted fabric structures.

## Figures and Tables

**Table 1:** Specifications of the 10 types of 100% cotton yarn used.

Type of cotton fibers	Yarn count (Tex)	Spinning method
Combed cotton	20	Conventional ring spinning
Combed cotton	15	Conventional ring spinning
Combed Supima cotton	20	Conventional ring spinning
Combed Supima cotton	15	Conventional ring spinning
Combed Supima cotton	12	Conventional ring spinning
Combed Supima cotton	10	Conventional ring spinning
Combed Supima cotton ESTex	20	Torque-free ring spinning
Combed Supima cotton ESTex	15	Torque-free ring spinning
Combed Supima cotton ESTex	12	Torque-free ring spinning
Combed Supima cotton ESTex	10	Torque-free ring spinning

**Table 2:** Notations and types of stitches of the 9 single knitted structures [37].

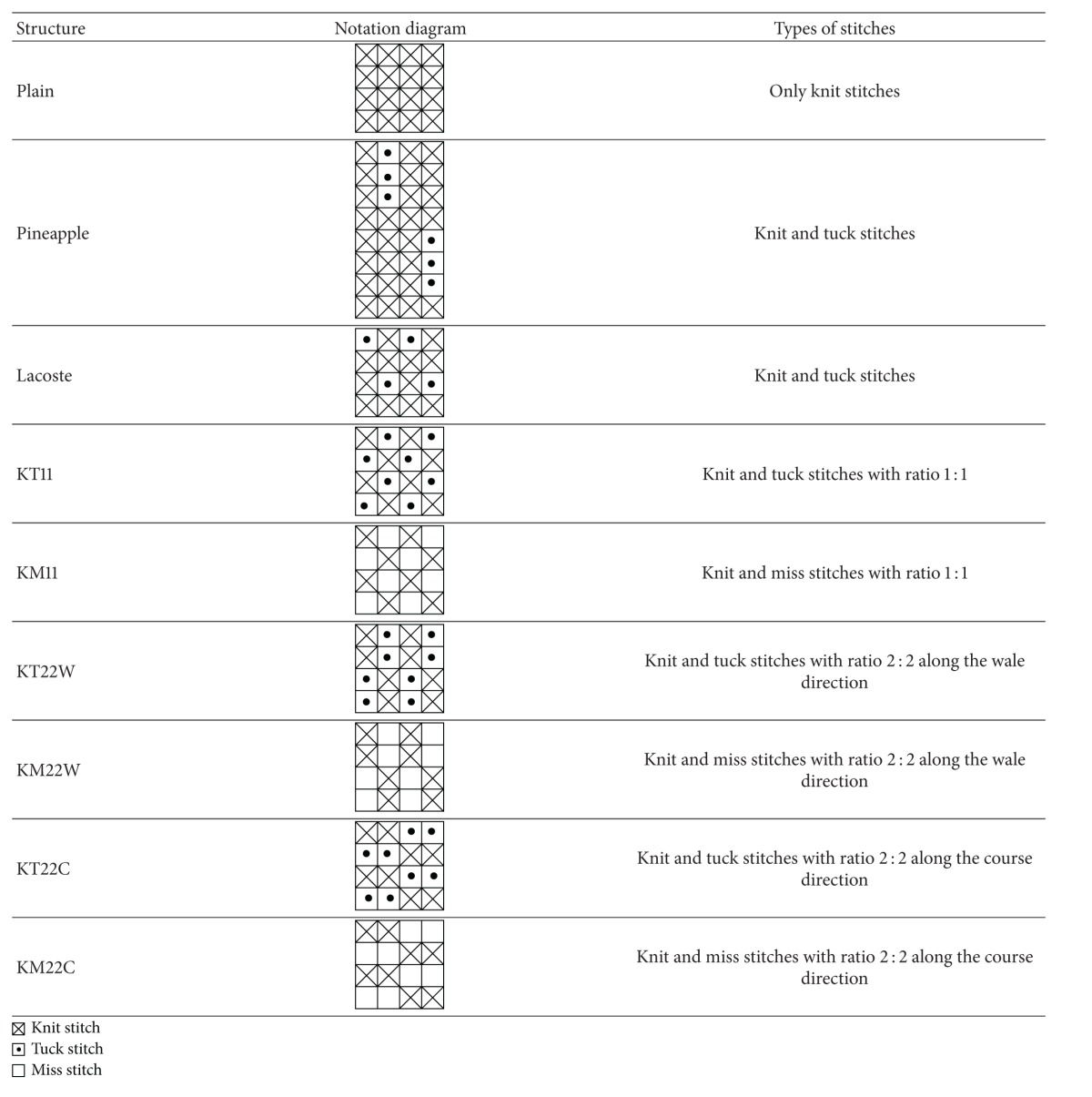

**Table 3:** Notations and types of stitches of the 6 double knitted structures [37].

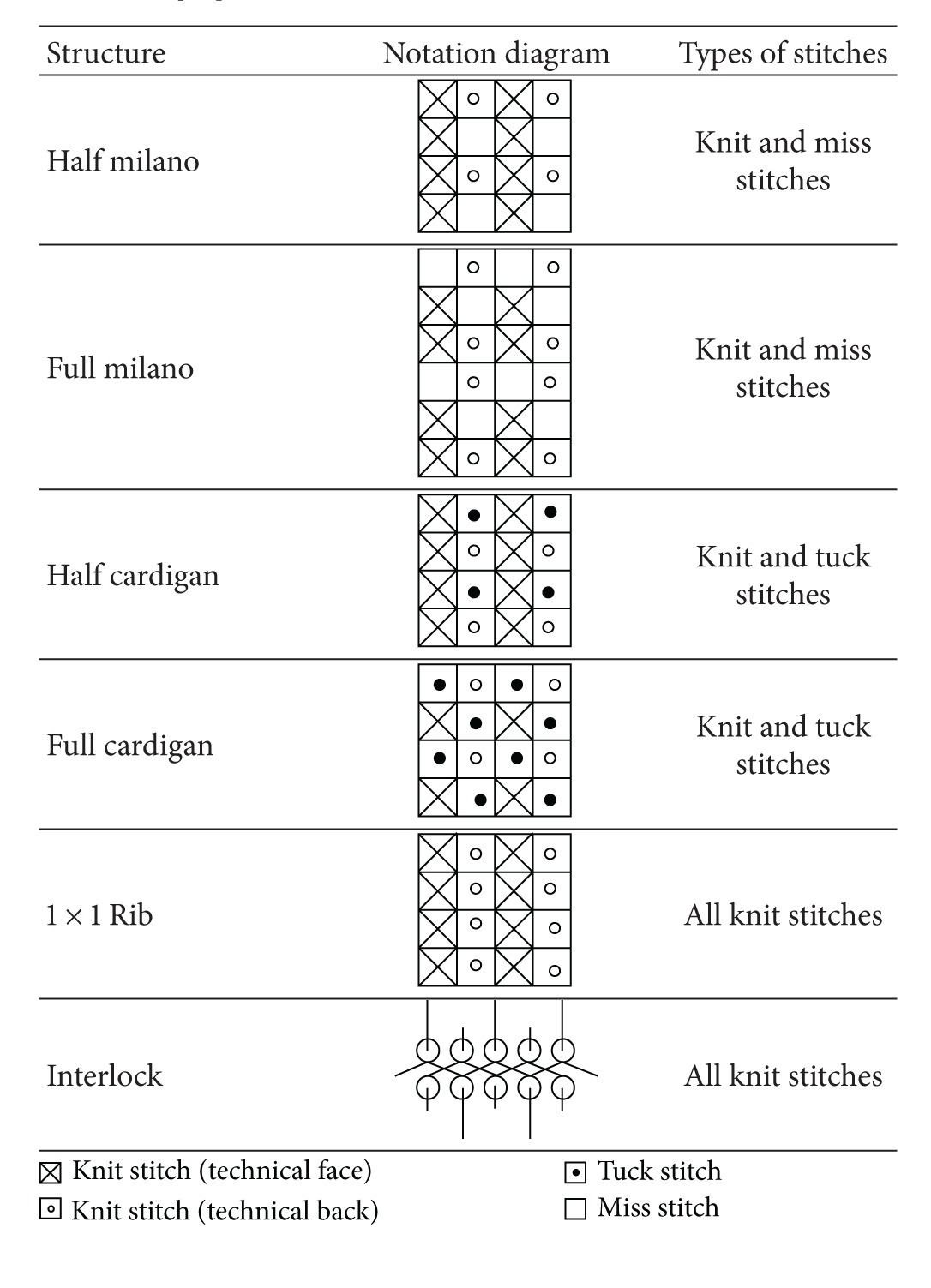

**Table 4:** Interpretation of Correlation Coefficient [35].

Correlation coefficient value (*R*)	Direction and strength of correlation
−1.0	Perfectly negative
−0.8	Strongly negative
−0.5	Moderately negative
−0.2	Weakly negative
0.0	No association
0.2	Weakly positive
0.5	Moderately positive
0.8	Strongly positive
1.0	Perfectly positive

**Table 5:** Relationship of UPF against fabric weight.

Structure	Nature of knitted structure	Types of stitches	Correlation coefficient (*R*)	Coffeicient of determination (*R* ^2^)	Mean fabric weight* (g/m^2^)	Mean UPF**
Plain	Single knit	Only knit	0.28	0.08	152.95	7.55
Pineapple	Single knit	Knit and tuck	0.16	0.02	159.46	8.07
Lacoste	Single knit	Knit and tuck	0.58	0.33	157.06	9.16
KT11	Single knit	Knit and tuck	0.83	0.70	146.53	7.86
KM11	Single knit	Knit and miss	0.72	0.52	181.64	14.42
KT22W	Single knit	Knit and tuck	0.67	0.45	133.25	6.29
KM22W	Single knit	Knit and miss	0.70	0.49	191.32	18.12
KT22C	Single knit	Knit and tuck	0.72	0.52	153.50	8.36
KM22C	Single knit	Knit and miss	0.97	0.94	177.11	15.25
Half milano	Double knit	Knit and miss	−0.15	0.02	211.85	24.21
Full milano	Double knit	Knit and miss	0.76	0.58	263.44	39.25
Half cardigan	Double knit	Knit and tuck	0.94	0.89	232.06	22.76
Full cardigan	Double knit	Knit and tuck	0.53	0.28	203.68	15.27
1 × 1 rib	Double knit	Only knit	0.44	0.19	197.95	20.00
Interlock	Double knit	Only knit	0.67	0.45	316.44	108.57
Single knitted structure^∧^			0.95	0.91		
Double knitted structure^∧∧^			0.94	0.88		

*Mean fabric weight refers to the average fabric weight of all yarn types in term of fabric structure.

**Mean UPF refers to the average of UPF all yarn types in term of fabric structure.

^∧^Statistical relationship for all single knitted structures (plain, pineapple, lacoste, KT11, KM11, KT22W, KM22W, KT22C, and KM22C) with all yarn types.

^∧∧^Statistical relationship for all double knitted structures (half milano, full milano, half cardigan, full cardigan, 1 × 1 rib, and interlock) with all yarn types.

**Table 6:** Relationship of UPF against fabric thickness.

Structure	Nature of knitted structure	Types of stitches	Correlation coefficient (*R*)	Coffeicient of determination (*R* ^2^)	Mean fabric thickness (mm)	Mean UPF**
Plain	Single knit	Only knit	0.23	0.05	0.99	7.55
Pineapple	Single knit	Knit and tuck	0.00	0.00	1.21	8.07
Lacoste	Single knit	Knit and tuck	0.42	0.18	1.34	9.16
KT11	Single knit	Knit and tuck	0.64	0.40	1.11	7.86
KM11	Single knit	Knit and miss	0.59	0.35	1.31	14.42
KT22W	Single knit	Knit and tuck	0.59	0.34	1.32	6.29
KM22W	Single knit	Knit and miss	0.63	0.39	1.43	18.12
KT22C	Single knit	Knit and tuck	0.55	0.30	1.26	8.36
KM22C	Single knit	Knit and miss	0.71	0.51	1.34	15.25
Half milano	Double knit	Knit and miss	0.41	0.16	1.52	24.21
Full milano	Double knit	Knit and miss	0.27	0.07	1.56	39.25
Half cardigan	Double knit	Knit and tuck	0.60	0.36	1.68	22.76
Full cardigan	Double knit	Knit and tuck	0.54	0.29	1.64	15.27
1 × 1 rib	Double knit	Only knit	0.57	0.32	1.46	20.00
Interlock	Double knit	Only knit	0.13	0.02	1.66	108.57
Single knitted structure^∧^			0.62	0.38		
Double knitted structure^∧∧^			0.37	0.13		

*Mean fabric thickness refers to the average fabric thickness of all yarn types in term of fabric structure.

**Mean UPF refers to the average of UPF all yarn types in term of fabric structure.

^∧^Statistical relationship for all single knitted structures (plain, pineapple, lacoste, KT11, KM11, KT22W, KM22W, KT22C, and KM22C) with all yarn types.

^∧∧^Statistical relationship for all double knitted structures (half milano, full milano, half cardigan, full cardigan, 1 × 1 rib, and interlock) with all yarn types.

**Table 7:** Relationship of UPF against *W*/*T* ratio.

Structure	Nature of knitted structure	Types of stitches	Correlation coefficient (*R*)	Coffeicient of determination (*R* ^2^)	Mean *W*/*T* ratio*	Mean UPF**
Plain	Single knit	Only knit	0.22	0.05	154.75	7.55
Pineapple	Single knit	Knit and tuck	0.30	0.09	132.04	8.07
Lacoste	Single knit	Knit and tuck	0.44	0.19	117.35	9.16
KT11	Single knit	Knit and tuck	0.42	0.17	132.84	7.86
KM11	Single knit	Knit and miss	0.53	0.28	138.67	14.42
KT22W	Single knit	Knit and tuck	0.44	0.19	100.51	6.29
KM22W	Single knit	Knit and miss	0.59	0.35	133.11	18.12
KT22C	Single knit	Knit and tuck	0.55	0.30	122.09	8.36
KM22C	Single knit	Knit and miss	0.82	0.68	132.34	15.25
Half milano	Double knit	Knit and miss	0.38	0.14	139.08	24.21
Full milano	Double knit	Knit and miss	0.82	0.67	168.44	39.25
Half cardigan	Double knit	Knit and tuck	0.70	0.48	137.56	22.76
Full cardigan	Double knit	Knit and tuck	0.21	0.04	124.17	15.27
1 × 1 rib	Double knit	Only knit	−0.19	0.03	135.44	20.00
Interlock	Double knit	Only knit	0.73	0.53	190.73	108.57
Single knitted structure^∧^			0.28	0.08		
Double knitted structure^∧∧^			0.92	0.85		

*Mean *W*/*T* ratio refers to the average *W*/*T* ratio of all yarn types in term of fabric structure.

**Mean UPF refers to the average of UPF all yarn types in term of fabric structure.

^∧^Statistical relationship for all single knitted structures (plain, pineapple, lacoste, KT11, KM11, KT22W, KM22W, KT22C, and KM22C) with all yarn types.

^∧∧^Statistical relationship for all double knitted structures (half milano, full milano, half cardigan, full cardigan, 1 × 1 rib, and interlock) with all yarn types.

**Table 8:** Relationship of UPF against stitch density.

Structure	Nature of knitted structure	Types of stitches	Correlation coefficient (*R*)	Coffeicient of determination (*R* ^2^)	Mean stitch density*	Mean UPF**
Plain	Single knit	Only knit	0.19	0.04	621.1	7.55
Pineapple	Single knit	Knit and tuck	0.71	0.50	529	8.07
Lacoste	Single knit	Knit and tuck	0.23	0.05	506.2	9.16
KT11	Single knit	Knit and tuck	0.37	0.14	303.7	7.86
KM11	Single knit	Knit and miss	0.23	0.05	553.7	14.42
KT22W	Single knit	Knit and tuck	0.06	0.00	287.4	6.29
KM22W	Single knit	Knit and miss	0.12	0.01	618	18.12
KT22C	Single knit	Knit and tuck	−0.06	0.00	315.7	8.36
KM22C	Single knit	Knit and miss	0.36	0.13	563.1	15.25
Half milano	Double knit	Knit and miss	0.38	0.14	505.9	24.21
Full milano	Double knit	Knit and miss	0.13	0.02	579.6	39.25
Half cardigan	Double knit	Knit and tuck	0.80	0.63	288.3	22.76
Full cardigan	Double knit	Knit and tuck	0.29	0.09	255.4	15.27
1 × 1 rib	Double knit	Only knit	−0.53	0.28	413.8	20.00
Interlock	Double knit	Only knit	0.23	0.05	717	108.57
Single knitted structure^∧^			0.61	0.37		
Double knitted structure^∧∧^			0.83	0.68		

*Mean stitch density refers to the average stitch density of all yarn types in term of fabric structure.

**Mean UPF refers to the average of UPF all yarn types in term of fabric structure.

^∧^Statistical relationship for all single knitted structures (plain, pineapple, lacoste, KT11, KM11, KT22W, KM22W, KT22C, and KM22C) with all yarn types.

^∧∧^Statistical relationship for all double knitted structures (half milano, full milano, half cardigan, full cardigan, 1 × 1 rib, and interlock) with all yarn types.
